# Construction, Verification and Experimental Use of Two Epitope-Tagged Collections of Budding Yeast Strains

**DOI:** 10.1002/cfg.449

**Published:** 2005

**Authors:** Russell Howson, Won-Ki Huh, Sina Ghaemmaghami, James V. Falvo, Kiowa Bower, Archana Belle, Noah Dephoure, Dennis D. Wykoff, Jonathan S. Weissman, Erin K. O'Shea

**Affiliations:** 1 Department of Biochemistry and Biophysics Howard Hughes Medical Institute University of California at San Francisco 600 16th Street, Genentech Hall, Room GH-S472D San Francisco CA 94143-2240 USA; 2 Cellular and Molecular Pharmacology Howard Hughes Medical Institute University of California at San Francisco 600 16th Street, Genentech Hall, Room GH-S472D San Francisco CA 94143-2240 USA; 3 School of Biological Sciences Seoul National University Seoul 151-742 Korea

## Abstract

A major challenge in the post-genomic era is the development of experimental approaches to monitor the properties of proteins on a proteome-wide level. It would be particularly useful to systematically assay protein subcellular localization, post-translational modifications and protein–protein interactions, both at steady state and in response to environmental stimuli. Development of new reagents and methods will enhance our ability to do so efficiently and systematically. Here we describe the construction of two collections of budding yeast strains that facilitate proteome-wide measurements of protein properties. These collections consist of strains with an epitope tag integrated at the C-terminus of essentially every open reading frame (ORF), one with the tandem affinity purification (TAP) tag, and one with the green fluorescent protein (GFP) tag. We show that in both of these collections we have accurately tagged a high proportion of all ORFs (approximately 75% of the proteome) by confirming expression of the fusion proteins. Furthermore, we demonstrate the use of the TAP collection in performing high-throughput immunoprecipitation experiments. Building on these collections and the methods described in this paper, we hope that the yeast community will expand both the quantity and type of proteome level data available.


**Comparative and Functional Genomics**
*Comp Funct Genom* 2005; **6**: 2–16.
Published online in Wiley InterScience (www.interscience.wiley.com). **DOI:** 10.1002/cfg.449

**Research Paper**

### Construction, verification and experimental use of two epitope-tagged collections of budding yeast strains

Russell Howson[1], Won-Ki Huh[1#], Sina Ghaemmaghami[2], James V. Falvo[1], Kiowa Bower[2], Archana Belle[1], Noah Dephoure[1], Dennis D. Wykoff[1], Jonathan S. Weissman[2] and Erin K. O'Shea[1]*

[1] *Department of Biochemistry and Biophysics, Howard Hughes Medical Institute, University of California at San Francisco, 600 16th Street, Genentech Hall, Room GH-S472D, San Francisco, CA 94143-2240, USA*
[2] *Cellular and Molecular Pharmacology, Howard Hughes Medical Institute, University of California at San Francisco, 600 16th Street, Genentech Hall, Room GH-S472D, San Francisco, CA 94143-2240, USA*

**Correspondence to: Erin K. O'Shea, Department of Biochemistry and Biophysics, Howard Hughes Medical Institute, University of California at San Francisco, 600 16th Street, Genentech Hall, Room GH-S472D, San Francisco, CA 94143-2240, USA. E-mail: oshea@biochem.ucsf.edu*

*# Present address: School of Biological Sciences, Seoul National University, Seoul 151-742, Republic of Korea.*

Revised: 18 November 2004
Accepted: 30 November 2004

### Abstract

**A major challenge in the post-genomic era is the development of experimental approaches to monitor the properties of proteins on a proteome-wide level. It would be particularly useful to systematically assay protein subcellular localization, post-translational modifications and protein–protein interactions, both at steady state and in response to environmental stimuli. Development of new reagents and methods will enhance our ability to do so efficiently and systematically. Here we describe the construction of two collections of budding yeast strains that facilitate proteome-wide measurements of protein properties. These collections consist of strains with an epitope tag integrated at the C-terminus of essentially every open reading frame (ORF), one with the tandem affinity purification (TAP) tag, and one with the green fluorescent protein (GFP) tag. We show that in both of these collections we have accurately tagged a high proportion of all ORFs (approximately 75% of the proteome) by confirming expression of the fusion proteins. Furthermore, we demonstrate the use of the TAP collection in performing high-throughput immunoprecipitation experiments. Building on these collections and the methods described in this paper, we hope that the yeast community will expand both the quantity and type of proteome level data available. Copyright © 2005 John Wiley & Sons, Ltd.**

**Keywords:** **tandem affinity purification; green fluorescent protein; epitope tagging; protein localization; protein expression; immunoprecipitation; proteomics**

**Supplementary material for this article can be found at http://www.interscience.wiley.com/jpages/1531–6912/suppmat**

### Introduction

The complete sequencing of the *Saccharomyces cerevisiae* genome in 1996 (Goffeau *et al.*, 1997) enabled a new era of global biological analysis of this organism. Sequence analysis of the genome has provided a wealth of information relevant to many aspects of yeast biology, most recently in comparison to the genomes of other yeast species (Brachat *et al.*, 2003; Cliften *et al.*, 2003; Kellis *et al.*, 2003). The development of whole genome transcriptional profiling using DNA microarrays has provided tools for assessment of the global transcriptional profile under different experimental conditions (DeRisi *et al.*, 1997; Lockhart *et al.*, 1996; Schena *et al.*, 1995). This approach has been extremely effective in understanding many biological processes.

There are, however, limitations to microarray analysis. Although the transcriptional profile of

Copyright © 2005 John Wiley & Sons, Ltd.

an organism is informative, many biological processes do not produce readily interpretable transcriptional readouts. Additionally, the protein effectors of biological processes in the cell cannot be directly monitored through the transcriptional profile. It would be particularly informative to be able to systematically monitor post-translational modifications, localization and protein–protein interactions in order to understand how the dynamic properties of proteins allow them to carry out biological processes.

Part of what makes microarray analysis possible is the chemical similarity and stability of nucleic acids. Despite the fact that these genes encode proteins of diverse composition, structure and function, the associated nucleic acids have virtually identical chemical properties. As a result, chemical manipulations can be done for all genes in parallel, simply by separating the nucleic acids spatially.

The same type of global analysis has been extremely challenging to achieve for proteins. The diversity of protein structure, chemical composition and stability makes generalized manipulation difficult. Further, the behaviour of proteins in isolation is frequently not identical to their activity in the context of the cellular milieu. Mass spectrometry and two-dimensional electrophoresis have been used with some success in global protein analysis (Aebersold and Mann, 2003), but have been hampered by the complexity of the proteome, and thus far have not been capable of analyses of a truly global nature. In addition, these techniques have a somewhat limited scope in the types of protein characteristics they are capable of measuring. Some groups have created purified protein libraries (Martzen *et al*., 1999) or protein microarrays (MacBeath and Schreiber, 2000; Phizicky *et al*., 2003; Zhu *et al*., 2001), but these approaches have suffered from both the significant effort involved in purifying the proteins and the inherent caveats of working with proteins *in vitro*. A further problem that has plagued all of these approaches has been difficulty in accurately assessing coverage of the proteome.

It would therefore be valuable to have a system that enabled systematic high-throughput analysis of the yeast proteome, both *in vivo* and *in vitro*. One could construct such a system by fusing a constant epitope tag to all proteins, in essence making these proteins more chemically similar to each other. Such similarity would enable systematic manipulation, purification or analysis of these proteins by a single method.

Here we describe the design and synthesis of a set of oligonucleotide primers useful for the genomic integration of DNA coding for any epitope tag at the C-terminus of every ORF in the yeast genome. Further, we describe the construction of two collections of yeast strains, one with the TAP tag and one with GFP tag, and the high-throughput technology and methodology that enabled the construction of these collections by a small team in a relatively short period of time. Lastly, we discuss the utility of these collections in the systematic execution of high-throughput biochemical and microscopic assays of the yeast proteome.

### Materials and methods

### Use of robotics and other high-throughput tools

In designing the oligonucleotides and the collections, as well as in developing the methodology for the creation and use of these collections, we made every effort to utilize available high-throughput technologies. The collection was designed in 96-well format to enable efficient oligonucleotide synthesis using a 96-well DNA synthesizer (GeneMachines Polyplex) and subsequent liquid handling by a robotic 96-well pipettor (Beckman Biomek FX). The Biomek FX was used for almost all liquid handling applications, including resuspending oligonucleotides, setting up all PCRs, loading agarose gels, transformations, inoculating cultures and adding lysis buffer to cell pellets. In cases where the Biomek FX could not be used, we instead employed electronic multichannel pipettors, for applications such as loading SDS-PAGE gels and dispensing buffers. In minimizing the number of manual steps performed, we were able to both optimize the efficiency of construction and minimize the opportunity for human error. This enabled the entire process to be completed in-house by a relatively small team.

### Oligonucleotide primer design and synthesis

The yeast genome sequence, as well as the coordinates of all ORFs, were obtained by download from the *Saccharomyces* Genome Database

Copyright © 2005 John Wiley & Sons, Ltd. 

(Dolinksi *et al.*; **ftp://ftp.yeastgenome.org/yeast/**) on 17 April 2001. We removed all mitochondrial genes, as well as those encoding Ty elements. We then divided the ORFs into two categories: soluble and putative membrane proteins, reasoning that having membrane proteins as a separate group would facilitate any modifications in biochemical assays needed for these proteins. The following criteria were used to put ORFs into the putative membrane category: (a) any protein experimentally determined to be an integral membrane or membrane-associated protein; (b) any protein with homology to a membrane or membrane-associated protein; and (c) any protein with $\geqslant 2$ putative transmembrane domains which appeared in the microarray data of polysome-associated RNAs (Diehn *et al.*, 2000).

Within the soluble and membrane categories, we ordered genes by size with the largest first, and then divided ORFs into groups of 96 to facilitate subsequent manipulations in 96- and 384-well plates. Each ORF is designated by a plate number and coordinates within that plate. All reagents and strains relevant to a given ORF occupy the same unique coordinates. This facilitated subsequent manipulations by the Biomek FX.

We used the 'Promoter' program (courtesy of Joe DeRisi, publicly available at: **http://derisilab.ucsf.edu**) to extract the last 40 nucleotides (excluding the stop codon) of each ORF, as well as 40 nucleotides of genomic sequence immediately following the stop codon of each ORF. We added the constant forward sequence from the 'Pringle' oligonucleotide-directed homologous recombination system (Longtine *et al.*, 1998) to the last 40 nucleotides of each ORF to create the F2 oligo sequence, and the reverse complement of the 40 nucleotides following each ORF to the constant reverse sequence to create the R1 oligo sequence. To design the sequence of the unique check primer for each ORF, we utilized Primer 3.0 (Rozen and Skaletsky; **http://www-genome.wi.mit.edu/genome_software/other/primer3.html**), selecting oligonucleotide primers with melting temperatures of 60 °C and which hybridize at 400–650 nucleotides upstream of the stop codon for each ORF. We note that this approach was used for all ORFs, including those representing repeated genes. The oligonucleotide sequences are available in the Supplementary Materials (**http://www.interscience.wiley.com/jpages/1531-6912/suppmat**), and also can be downloaded from the website accompanying the research papers (**http://yeastgfp.ucsf.edu**).

Oligonucleotides were synthesized on a GeneMachines Polyplex 96-well oligonucleotide synthesizer, which was modified to accommodate larger reagent bottles required for 60-mer synthesis. All DNA synthesis reagents used were from Glen Research. We used a protocol (see Supplementary Materials; **http://www.interscience.wiley.com/jpages/1531-6912/suppmat**) optimized for producing full length 60-mers without a need for changing reagent bottles (involving more and longer coupling steps with less volume), enabling us to run the machine overnight, which allowed for the efficient synthesis of the 12 468 60-base and 6234 20-base oligonucleotides required.

Synthesized oligonucleotides were cleaved off the solid synthesis support by incubating three times with 100 µl $NH_4OH$ for 10 min, followed by collection into a deep well 96-well plate with a vacuum manifold (Millipore). The oligos were then deprotected by heating at 65 °C for 15–24 h, and lyophilized in a Sorvall SpeedVac AES2010 to remove the $NH_4OH$. Prior to use, oligonucleotides were resuspended to a concentration of 100 µM with deionized water (typically 200–300 µl, depending on the scale of DNA synthesis).

### Construction of collections

We performed PCR and transformation in 96-well format as follows: F2 and R1 oligos were combined to working concentrations of 5 µM each. 10 µl of the primer combination were added to 40 µl of a PCR mix [19.5 µl $H_2O$, 5 µl 10× Pwo buffer, 5 µl 20 mM $MgCl_2$, 5 µl 2 mM dNTPs, 5 µl plasmid template DNA (∼2 ng/ml), 0.5 µl Expand DNA polymerase] aliquotted to 96-well microplates in order to amplify the desired tag. For PCR, we used an MJ Research Tetrad thermal cycler with the following program: 94 °C 3 min, 10× (94 °C 15 s, 50 °C 30 s, 72 °C 2 min), 15× (94 °C 15 s, 72 °C 2 min + 5 s/cycle), 72 °C 10 min. Following PCR, we checked for a product of correct size using 96-well agarose gels (Amersham Ready to Run system) loaded with the Biomek FX.

These PCR products were then transformed into our base strain (ATCC #201 388: S288C,

Copyright © 2005 John Wiley & Sons, Ltd.

*MAT***a** *his3Δ1 leu2Δ0 met15Δ0 ura3Δ0*; Brachmann *et al.*, 1998). Cells were grown to $OD_{600}$ of 0.7–0.8 in YEPD; each transformation in the 96-well plate required 3 ml of starting culture. After being pelleted at $\sim 1000 \times g$ at room temperature, cells were washed twice in 1/10 of the original culture volume of 100 mM lithium acetate and resuspended in 1/100 of the original culture volume of 100 mM lithium acetate. For each transformation, we added 15 μl unpurified PCR product to 183 μl aliquots of the following transformation recipe, mixed by vortexing at temperature and scaled appropriately for 96-well format: 100 μl 50% weight/volume PEG (MW 3350, freshly prepared and filtered at 0.2 μm), 15 μl 1 M lithium acetate, 20 μl salmon sperm carrier DNA (2 mg/ml), 18 μl DMSO, and 30 μl yeast cell suspension. We then performed incubations (30 °C for 30 min, followed by 42 °C for 15 min) for transformation in the thermal cycler. Cells were pelleted in the microplates at $\sim 1000 \times g$ at room temperature, resuspended in 100 μl water, and plated manually on standard yeast synthetic medium plates lacking histidine (SD–His) to select for genomic integrants.

After growth for 3 days, transformations typically yielded 5–100 colonies. We selected up to six individual transformants for each ORF and streaked onto fresh selective medium. After subsequent growth, we performed whole-cell PCR on each transformant to determine whether the tag had integrated at the correct locus. A small aliquot of freshly grown cells was resuspended in 5 μl water and boiled in 96-well format (99 °C for 5 min in the thermal cycler). 5 μl boiled cells and 2.5 μl 5 μM unique 'check' oligos were added to PCR mix (13 μl $H_2O$, 2.5 μl 10× Taq buffer, 1.5 μl 2 mM dNTPs, 0.25 μl 50 μM 'F2CHK' primer, 0.25 μl 5U/μl Taq Polymerase, 0.05 μl 10 mg/ml RNase) and PCR was performed [94 °C 2.5 min, 35× (94 °C 45 s, 55 °C 45 s, 72 °C 1 min), 72 °C 10 min]. We analysed the results of these PCRs by 96-well agarose gel electrophoresis, identifying correct integrants by the presence of a PCR product of appropriate size.

For construction of the GFP collection, we used much the same method, except individual transformants were picked and directly inoculated into 600 μl SD-His medium for overnight growth. We centrifuged 200 μl of these cultures in 96-well PCR plates to pellet cells, removed the supernatant, and lysed the cells in 20 μl 0.2% SDS at 99 °C for 10 min in the PCR machine. We then used 0.6 μl of this lysate as template for a 20 μl PCR (16.2 μl $H_2O$, 2 μl 10× Taq buffer, 0.6 μl 2 mM dNTPs, 0.2 μl each 50 μM oligonucleotide primer, 0.2 μl 5 U/μl Taq polymerase, 0.04 μl 10 mg/ml RNAse) to confirm correct integration of the tag. The presence of a PCR product was analysed by 96-well agarose gel electrophoresis.

### Assembly and growth

To assemble these strain collections into 96-well plates, we selected two correct integrants (when possible) for each ORF and resuspended cells in 0.5× SD-His +15% glycerol. The plate number and coordinates were maintained for each ORF, resulting in an 'A' and 'B' collection for each set of epitope-tagged strains, which we froze at −80 °C. For the GFP strains, we assembled the A and B collections directly from the liquid cultures used for confirmation PCR, mixing saturated cultures with 30% glycerol and freezing.

Subsequent growth was achieved by thawing the glycerol stocks and either inoculating liquid cultures with a Biomek FX robot or spotting onto YEPD plates with a 96-well pinning tool. To grow these cultures in high-throughput format, we used a GeneMachines HiGro growth chamber. Typically, cultures were 1.5–2.0 ml, and cells were grown at 30 °C and 500 rpm. Under these conditions, cells grew with the same growth rate as liquid cultures in standard flasks (data not shown). We sometimes used Teflon-coated magnetic beads in each well to enhance mixing of the culture. Addition of these beads did not have an effect on growth rate (data not shown), but did prevent settling of cells that began to occur in late log phase.

### Immunoblot analysis of the TAP collection

To analyse the TAP collection by immunoblotting, 200 μl aliquots of YEPD medium in a 96 well plate were inoculated with cells from a plate with a 96-well pinning tool and allowed to grow to saturation overnight. We diluted these cultures to $OD_{600} = \sim 0.1$ into 1.8 ml YEPD in 2 ml deep-well 96-well plates and grew them to logarithmic phase ($0.8 < OD_{600} < 1.0$) at 30 °C and 500 rpm in a GeneMachines HiGro Shaker. Log phase cultures were centrifuged to pellet the yeast cells.

Copyright © 2005 John Wiley & Sons, Ltd.

We removed the medium supernatant and added 50 μl hot SDS lysis buffer (50 mM Tris, pH 7.5, 5% SDS, 5% glycerol, 50 mM DTT, 5 mM EDTA, bromophenol blue, 2 μg/ml leupeptin, 2 μg/ml pepstatin A, 1 μg/ml chymostatin, 0.15 mg/ml benzamidine, 0.1 mg/ml pefabloc, 8.8 μg/ml aprotinin, 3 μg/ml anipatin) and boiled (99 °C for 10 min in the thermal cycler). These lysates were centrifuged, and the supernatant was kept and frozen at −80 °C.

We loaded 13 μl of these lysates on 26-well 4–15% gradient precast Criterion gels (Bio-Rad) with a multi-channel pipettor (Matrix Technologies Impact2). Gels were run and transferred to PVDF membranes with a Transblot SD semi-dry blotter (Bio-Rad). Immunoblot analysis was performed using a primary antibody mixture of a rabbit polyclonal affinity-purified antibody to the calmodulin-binding peptide (CBP) portion of the TAP tag (1 : 5000 dilution) and an anti-hexokinase antibody (US Biological, 1 : 50 000) as a loading control and quantitation standard. A horseradish peroxidase (HRP) conjugated goat anti-rabbit antibody (Bio-Rad) was used as a secondary antibody, and the SuperSignal West Femto Maximum Sensitivity ECL substrate (Pierce) was used for detection. Images were collected with a CCD-based imaging system (Alpha Innotech Fluorochem 8800) and analysed with the FluoroChem FC software (Alpha Innotech).

### Fluorescence microscopic analysis of the GFP collection

We grew cells from the GFP collection to log phase in the same way, except in SD-His medium. Aliquots of these cultures [100 μl of a 1 : 10 dilution in SD-His with a final concentration of 1 μg/ml 4′,6-diamidino-2-phenylindole (DAPI)] were analysed in 96-well glass-bottomed microscope slides (BD Falcon #357 311) pre-treated with concanavalin A (50 μg/ml in water) to ensure cell adhesion. Prior to using the slides, we added 100 μl concanavalin A solution to each well, incubated at room temperature for at least 30 min, washed five times with distilled water, removed excess liquid by vigorous shaking, and dried the plates right-side up and covered by a lid overnight. We imaged cells using a Nikon TE200/300 inverted microscope with a 100× oil-immersion objective (the slide bottoms were painted with immersion oil), and made use of scripting functions in the MetaMorph version 4.6r8 imaging software in order to automate most of this process. GFP (Chroma filter set 41 020, exciter HQ480/20x, dichroic Q495LP, emitter HQ510/20m) and DAPI (Chroma filter set 86 010, exciter S375/20×, dichroic 86 010bs, emitter S415/24m) fluorescence images (2 s exposure), as well as differential interference contrast (DIC) images (10 ms exposure), were collected for each strain and analysed for expression and subcellular localization (Huh *et al*., 2003). For co-localization analysis (Huh *et al*., 2003), RFP was visualized using Chroma filter set 86 010, exciter S580/20×, dichroic 86 010bs, and emitter S630/60m and GFP was visualized using Chroma filter set 86 010, exciter S492/18x, dichroic 86 010bs, and emitter S530/40m; use of the same dichroic mirror minimized the time lapse between fluorescent images.

### Reorganization of TAP collection

With the TAP immunoblot data in hand (Ghaemaghammi *et al*., 2003), we reorganized the TAP collection strains according to abundance. Based on our measured protein expression levels, we divided the strains into five different expression level categories, designated GS1, GS2, GS3, GS4 and GS5. GS1 is the highest expression category and GS2, GS3 and GS4 have successively lower expression levels, with upper cutoffs of $3.5 \times 10^4$, $4.75 \times 10^3$ and $1.38 \times 10^3$ molecules/cell respectively. The GS5 category includes all ORFs that could be visualized on the blots but whose abundance could not be quantified, either because the expression level was close to background or there were other technical complications with the quantitation (e.g. the protein did not run primarily as a single band). Within each abundance category, the proteins are arranged based on predicted size from largest to smallest. The membrane proteins have been placed at the end of each category, also arranged from large to small. The organization of the TAP collection is available in the supplementary materials (**http://www.interscience.wiley.com/jpages/1531–6912/suppmat**).

### 96-well growth and native extract preparation

To grow cells for extract preparation, we first inoculated 600 μl YEPD liquid cultures from a YEPD plate, using a 96-well pinning tool. These cultures were allowed to grow to saturation overnight on the

Copyright © 2005 John Wiley & Sons, Ltd. 

benchtop with no agitation. The next morning we diluted these cultures into six deep-well 96-well plates, with 1.8 ml YEPD medium in each well, to an $OD_{600} = 0.1–0.2$, and grew to log phase ($0.8 < OD_{600} < 1.0$) at 30 °C and 500 rpm in a GeneMachines HiGro Shaker. When cultures had reached log phase, we centrifuged the plates at 3000 rpm for 10 min and aspirated the medium. The cell pellets were resuspended in 150 µl cold sorbitol buffer (1.2 M sorbitol, 0.1 M $KPO_4$, pH 7) +2 µl/ml 2-mercaptoethanol. We then combined the cell suspensions from six 96-well plates, maintaining the coordinates, and centrifuged and aspirated again. These pellets were then resuspended in 150 µl cold sorbitol buffer with 2 µl/ml 2-mercaptoethanol and 60 µl/ml lyticase (Haswell and O'Shea, 1999), and transferred to a 96-well PCR plate. We incubated these plates at 30 °C for 15 min in the thermal cycler, then centrifuged gently (1000 × *g*) for 10 min. After aspirating the supernatant, the pellets were washed gently in sorbitol buffer, frozen in liquid nitrogen and stored at −80 °C.

To lyse the cells, we resuspended the thawed cell pellets in 100 µl hypotonic lysis buffer (50 mM Tris, pH 7.5, 5 mM $MgCl_2$, 5 mM EGTA, 1 mM EDTA, 0.1% Triton X-100, 1 mM 2-mercaptoethanol, 2 mM PMSF, 2.5 mM benzamidine, 1 µg/ml leupeptin, 1 µg/ml pepstatin). After incubation on ice for 10 min, we added 20 µl buffer plus 0.9 M NaCl (to make the final buffer 150 mM NaCl) and incubated on ice for another 10 min. We then centrifuged at 4000 rpm in a Beckman RC-3B swinging bucket rotor fitted with 96-well plate carriers for 20 min to pellet cellular debris. Following centrifugation, we removed 100 µl lysate to a new 96-well plate containing 25 µl 50% glycerol in each well, and kept a small aliquot to measure protein concentration by Bradford assay. These extracts were frozen in liquid nitrogen and stored at −80 °C. Each well contained 125 µl total extract and typically were ∼10 mg/ml.

### Multiplexed immunoprecipitations

To perform high-throughput immunoprecipitations, we first prepared native extracts as above, except we combined cell pellets from six *different* cultures into one plate. The result is that each well contains a total of 125 µl ∼ 10 mg/ml extract, but derived from a mixture of six different TAP-tagged strains. We combined strains with approximately equal expression levels of the fusion protein in order to minimize dominance of well-expressed proteins or loss of minimally expressed proteins.

To perform the immunoprecipitation reactions, we first diluted extracts in 1 ml deep-well 96-well plates to a total volume of 560 µl with P buffer (50 mM Tris, pH 7.5, 150 mM NaCl, 5 mM $MgCl_2$, 5 mM EGTA, 1 mM EDTA, 0.1% Triton X-100, 1 mM 2-mercaptoethanol, 2 mM PMSF, 2.5 mM benzamidine, 1 µg/ml leupeptin, 1 µg/ml pepstatin). We then added 3 µg biotin-conjugated Human IgG (Jackson Immunoresearch), and incubated extracts for 30 min at 4 °C. 40 µl of a 25% suspension of streptavidin beads (Amersham Pharmacia) was then added to each well. We incubated the plate at 4 °C for another 30 min, vortexing *very gently* three or four times during this period to resuspend the beads.

We then transferred the reactions with a multichannel pipettor to a filter plate (Orochem catalogue no. OF1100). This type of plate contains 96 wells, each with a small frit above an opening at the bottom of each well. We placed this plate on a vacuum manifold; applying a vacuum allows for the removal of the supernatant but retention of the beads. Beads were washed four times with 400 µl of PDMS buffer (P buffer + 1% Triton X-100 + 300 mM NaCl). We then centrifuged the plate briefly (1000 × *g* for 1 min) to remove any residual liquid remaining in or around each well. 10 µl sample buffer was added to each well, and the plate was vortexed to resuspend the beads in the sample buffer. The plate was allowed to stand at room temperature for 5 min, and then centrifuged (1000 × *g* for 2 min) on top of a shallow 96-well plate to collect the eluate. This process was repeated again, to generate a total elution volume of 20 µl.

To analyse the results of the multiplex immunoprecipitation, the samples were run on 26-well Criterion SDS-PAGE gels (Bio-Rad), and transferred to nitrocellulose in 20 mM $NaPO_4$, pH 6.8, buffer with a BioRad Transblot apparatus. We performed immunoblot analysis by probing with Rabbit Fc (Jackson Immunoresearch; 1 : 10 000 dilution of 3.8 mg/ml stock in TBST + 5% non-fat dry milk), an HRP-conjugated goat anti-rabbit Fc secondary

Copyright © 2005 John Wiley & Sons, Ltd. 

antibody (Jackson Immunoresearch; 1 : 50 000 dilution of a 1 mg/ml stock in TBST + 5% milk), and the SuperSignal West Femto Maximum Sensitivity ECL substrate (Pierce). Images were collected with a CCD camera (Alpha Innotech Fluorochem 8800).

### Availability of TAP and GFP collections

The TAP collection is available from Open Biosystems (**http://www.openbiosystems.com**) and the GFP collection is available from Invitrogen (**http://www.invitrogen.com**).

### Results and discussion

### Collection design and oligonucleotide synthesis

To create a collection of tagged yeast strains, we utilized oligonucleotide directed homologous recombination (Figure 1A) (Longtine *et al*., 1998) to integrate an epitope tag at the C-terminus of each ORF. Briefly, 60-mer oligonucleotide primers (the 'F2' and 'R1' primers) are designed which contain both a variable sequence, homologous to the gene of interest, and a constant sequence, which enables PCR amplification of sequence coding for the desired tag and a nutritional marker used to select for integrants. Homologous sequences in the oligonucleotides direct integration of the tag at the desired location in the genome. Nutritional selection is used to isolate the integrants, and integration at the correct locus is then confirmed, using PCR with one primer in the tag and one specific to the targeted ORF (Figures 1B, 2A). We chose to tag the C-terminus of each ORF so that the endogenous promoter would remain intact, and to minimize the impact on the signal sequence of secreted and membrane proteins. We therefore designed and synthesized the required oligonucleotides for each ORF in the yeast genome (see Methods for details). Although we chose to construct two collections, one with the TAP tag and the other with the GFP tag, this oligonucleotide set could be used to construct a collection of strains with any desired tag integrated at the C-terminus of each ORF.

An important consideration in the design of the collections was the ability to take advantage of high-throughput and automation technologies. We designed the oligos in 96-well format to be efficiently synthesized by a high-throughput synthesizer, and developed methods for construction and use of the collections based around liquid handling robots and multi-channel pipettors. The use of these tools was imperative in making a project of this nature feasible. After refinement of these methods during construction of the TAP collection, the only manual steps performed in the construction of the GFP collection were plating transformations and picking colonies.

### Construction of the TAP and GFP collections

The first round of construction of the TAP collection was accomplished in about 4 months by a team of six people. We subsequently further refined the protocol for the confirmation PCR by growing individual transformants in liquid culture rather than on solid medium, enabling all manipulations to be performed by the Biomek FX liquid handling robot. With this refinement, as well as others, the efficiency of the construction process was greatly enhanced: the first round of construction of the GFP collection was carried out by two people in under 3 months. This oligo set and these methods could therefore be useful in the efficient construction of new collections tailored to particular experiments.

After the first round of construction of the TAP collection, we successfully tagged 97% of 6234 ORFs, as assayed by genomic PCR. For the GFP collection, we obtained PCR-positive clones for 99% of ORFs. We also determined that the frequency of obtaining a properly integrated strain (assayed by genomic PCR) was roughly equivalent between essential (93%) and non-essential (98%) ORFs.

### Confirmation of correctly expressed fusion proteins and reconstruction

We collected two PCR-positive clones (when possible) for each ORF, and assembled the strains into an 'A' and 'B' set for each collection. In beginning to work with these strains, we discovered some inconsistencies in expression of tagged proteins in different isolates from the same tagged ORF. Specifically, we identified some cases in which only one of the two isolates expressed the protein of interest, despite the fact that correct integration was confirmed for both by genomic PCR. After sequence analysis of representative isolates, we identified sequence errors in the

Copyright © 2005 John Wiley & Sons, Ltd. 

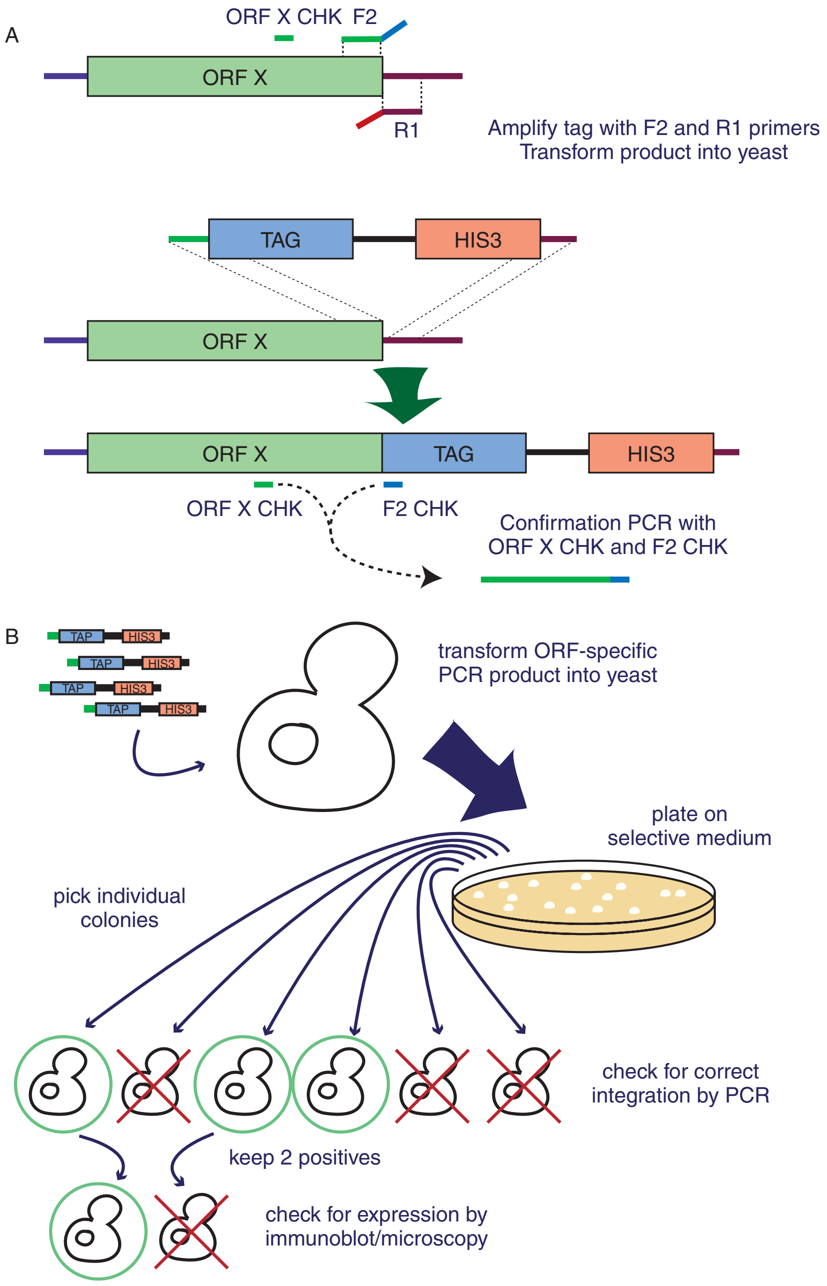

**Figure 1.** Schematic of construction and verification process for strain collections. (A) PCR-mediated homologous recombination. Three oligonucleotide primers are designed and synthesized for each ORF: the F2 and R1 oligos and a CHK oligo. The F2 and R1 oligos, containing regions of homology to the ORF of interest, are used to amplify the desired tag and a selectable marker; this PCR product then integrates into the genome at the C-terminus of the ORF of interest. The CHK oligo, as well as an oligo within the tag, is used to verify integration at the desired location. (B) Generalized construction process. We transformed the PCR-amplified tag into yeast, plated on selective medium, and then picked individual colonies and verified correct integration of the tag. Correct integrants were then screened for expression of the protein fusion by immunoblot or microscopy and a correctly expressing isolate was selected

Copyright © 2005 John Wiley & Sons, Ltd. 

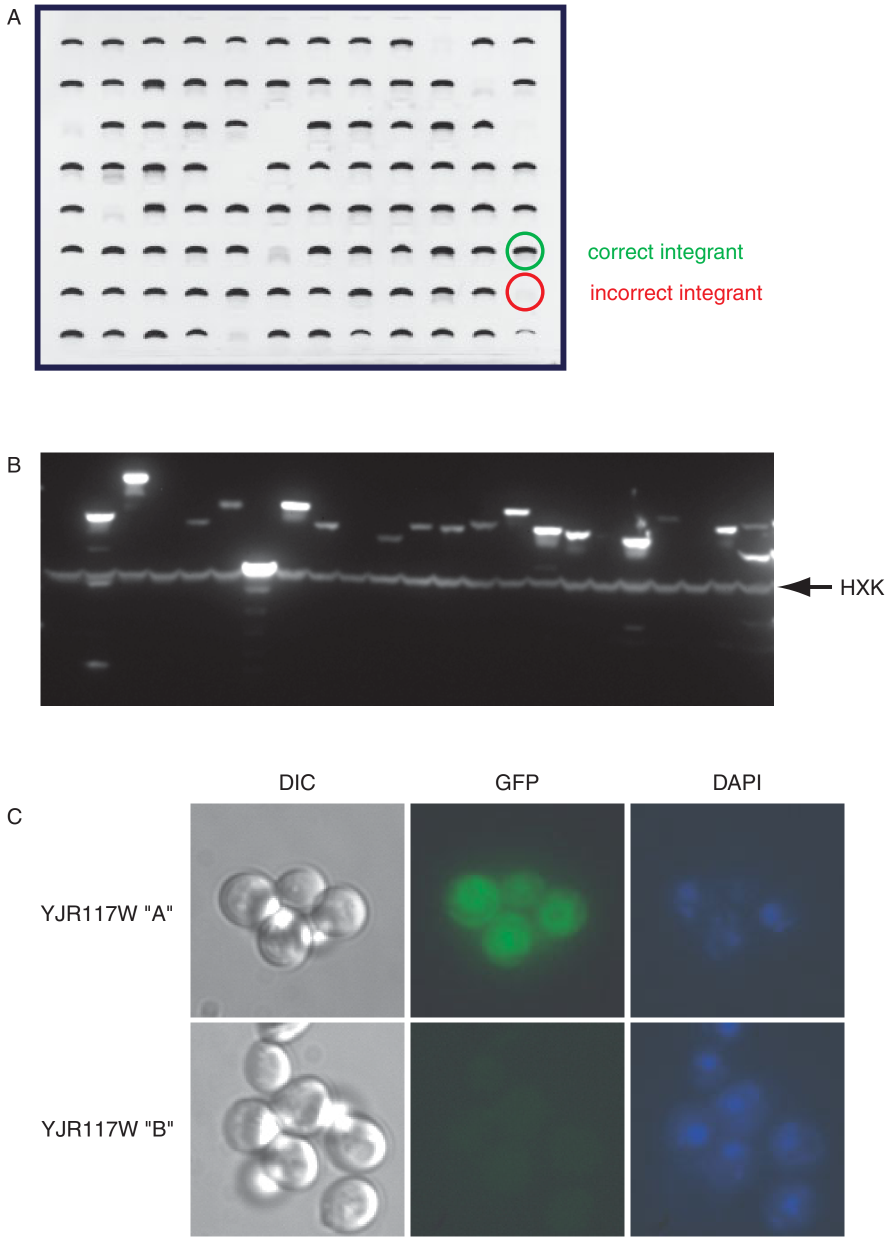

**Figure 2.** Examples of verification of integration and expression. (A) 96-Well agarose gel used to analyse check PCRs to confirm proper integration of the tag. Presence of band indicates proper integration, absence indicates improper integration. (B) Immunoblot analysis of TAP collection isolates. Arrow indicates the hexokinase band used as a loading control and quantitation standard. (C) Fluorescence microscopy of GFP collection isolates. Shown is an example in which expression from the A collection did not match expression from the B collection junction between the C-terminus of the protein and the tag, or in the tag itself. The source of these errors is presumably errors in the oligonucleotides themselves or in the tag amplification and transformation process. Regardless, we concluded that proper integration of the tag could only be reliably confirmed by analysing expression of the fusion protein (by immunoblot for the TAP collection, and fluorescence microscopy for the GFP collection).

Copyright © 2005 John Wiley & Sons, Ltd. 

We analysed log phase cultures from the A and B isolates of both collections for expression of the tagged proteins. For the TAP collection, we made SDS extracts and analysed these extracts by SDS-PAGE followed by immunoblotting against the TAP tag (Figure 2B). For the GFP collection, we observed the tagged proteins by fluorescence microscopy (Figure 2C). A more thorough discussion of these methods is in the Materials and methods section.

After this analysis, we were able to identify which isolates of a particular ORF were correctly expressing the tagged protein of interest. We found that the frequency of 'mistagged' ORFs was significant. In the TAP collection, 21% of PCR-positive 'B' isolates with a corresponding expression-positive A isolate yielded no detectable expression by immunoblot. This highlights the importance of expression analysis in confirming the accuracy of these collections.

In cases where expression could not be confirmed for either isolate, we were unable to distinguish between two possibilities. First, it is possible that neither isolate contained the correctly tagged ORF. Alternatively, the protein could be correctly tagged, but not expressed to detectable levels under our growth conditions. We therefore compared our results from both collections to determine if there were cases in which expression of a given ORF was detected in one collection but not the other. Given that these collections were constructed with the same oligo set, one would expect to obtain similar results with the two collections. Although these means of analysis are different and therefore may have different limits of detection, we reasoned that if a tagged protein was detected in one collection but not the other, it was probably not tagged correctly in the collection in which it was not detected.

With this information in hand, we undertook the task of reconstructing those strains (708 for the TAP collection, 759 for GFP) for which the ORF fusion was detected in one collection but not the other. In order to avoid again isolating strains that did not correctly express the tagged protein of interest, we omitted the confirmation PCR and instead analysed individual transformants by immunoblotting or microscopy. After this process, 457 new positives were obtained for the TAP collection, and 398 new positives were obtained for the GFP collection.

### Coverage of the proteome

In the construction of these collections, we monitored our success rate at many steps in order to assess the quality and utility of these collections in performing proteome-wide studies (Table 1). While we were able to obtain a 97% success rate by genetic analysis, our subsequent expression analysis indicated that this is not the most accurate metric for coverage of the proteome. Because of the potential for errors in the oligonucleotides and in the integration process, and the inability of genetic analysis to uncover these errors, we feel that detection of expression of the fusion proteins is the most accurate metric of coverage, especially considering that utility of these collections in monitoring protein characteristics ultimately rests on the ability to detect them.

By this metric, we detect 4251 proteins, or 68% of 6234 annotated ORFs, in the TAP collection, and 4156, or 67% of annotated ORFs, in the

**Table 1.** Details of success rate at various stages of construction for the TAP and GFP collections

| | TAP collection | | | | GFP collection | | | |
|---|---|---|---|---|---|---|---|---|
| | All ORFs | | Essential ORFs | | All ORFs | | Essential ORFs | |
| | # | % | # | % | # | % | # | % |
| Total ORFs | 6234 | 100 | 1100 | 100 | 6234 | 100 | 1100 | 100 |
| Tag amplified with ORF specific PCR | 6211 | 100 | 1096 | 100 | ND | | ND | |
| ≥1 Transformants obtained | 6047 | 97 | 1014 | 92 | 6151 | 99 | 1018 | 93 |
| Positive by check PCR | 6040 | 97 | 1003 | 92 | 6029 | 97 | 953 | 87 |
| Positive by expression (first round) | 3811 | 61 | 723 | 66 | 3758 | 60 | 712 | 65 |
| Positive by expression (after reconstruction) | 4251 | 68 | 821 | 75 | 4156 | 67 | 827 | 75 |

Success rates for all ORFs and essential ORFs of each collection are displayed, as well as the associated percentage. ND, not determined.

Copyright © 2005 John Wiley & Sons, Ltd. 

GFP collection (Ghaemmaghami *et al.*, 2003; Huh *et al.*, 2003). Previously, we estimated that 525 of the 6234 annotated ORFs are spurious (Ghaemmaghami *et al.*, 2003), a result similar to that derived from a comparative genomics study (Kellis *et al.*, 2003). These observations suggest that there are approximately 5700 protein-coding genes. Therefore, we observe ∼75% of the proteome by Western blot analysis of the TAP library and ∼73% of the proteome by fluorescence microscopic analysis of the GFP library. A total of 4517 ORFs, or 79% of the proteome, were detected in at least one collection (Ghaemmaghami *et al.*, 2003). The overlap between the proteins detected in these collections [over 90% of ORFs detected in the GFP collection were also detected in the TAP collection (Ghaemmaghami *et al.*, 2003; Huh *et al.*, 2003)] strengthens our conclusion that the collections represent a large majority of true protein-coding genes. These numbers will undoubtedly improve as more sensitive detection methods are developed (such as immunoprecipitation followed by immunoblot) and as expression in other growth conditions is examined.

However, some strains will certainly need to be reconstructed. We sequenced the ORF-tag junction and the tag for 35 strains for which the tagged ORF is known or strongly predicted to code for a protein, but the fusion protein was not detected in either collection. Of three clones for fusions of essential ORFs, two had mutations and one did not. Of five clones for fusions of ORFs with a high codon adaptation index (CAI), four had insertions or deletions and one had a non-synonymous substitution in the tag. Of 27 clones for fusions of ORFs coding for proteins with a previously reported localization (Dolinksi *et al.*; **ftp://ftp.yeastgenome.org/yeast/**), 20 had mutations while seven did not. It is possible that the oligonucleotide primers used to generate these strains contained more errors, or that some deleterious consequences of insertion of the tag caused selection against correct integrants.

If we take our success rate for essential proteins as a proxy of our overall success rate (since essential proteins are presumably true ORFs and expressed under normal growth conditions), our collections represent 75% of the proteome for the TAP collection and GFP collections. When looking at both collections, at least one fusion protein was detected for about 80% of essential ORFs. The high percentage of essential ORFs detected also indicates that the fusion protein is likely functional in a high proportion of cases. We conclude that these collections represent useful representations of the proteome for use in global analyses. In characterizing these collections, we were also able to make quantitative measurements of protein expression (Ghaemmaghami *et al.*, 2003) and describe cellular localization (Huh *et al.*, 2003) for the majority of the yeast proteome.

### ‘Multiplexed’ immunoprecipitations

In constructing these collections, we wished to not only be able to perform descriptive analyses, but also to do experiments systematically on the entire proteome. Our hope was that standard laboratory assays typically performed on a small number of strains or proteins could be applied systematically and efficiently to the entire proteome. The fact that every strain in the TAP collection utilizes the same tag enables a generalized method to be applied to all strains to perform large-scale experiments in parallel.

As a first step, we developed high-throughput methods to efficiently make extracts and immunoprecipitate proteins (Figure 3A). Briefly, this involves growing 2 ml cultures to log phase in 96-well format, combining cell pellets from six *different* 96-well plates, and spheroplasting cells with lyticase. We made extracts by osmotic lysis, pelleting cellular debris and keeping the supernatant. Each well of these ‘multiplex’ extracts contains extract from six different strains, and therefore six different TAP-tagged proteins, facilitating the parallel immunoprecipitation of six different proteins in each well of a 96-well plate. Immunoprecipitation in 96-well format, therefore, theoretically enables the simultaneous pulldown of 576 proteins.

Utilizing the quantitative immunoblot data obtained from screening the TAP collection (discussed above), we reorganized the TAP collection according to abundance. We divided the strains into six abundance categories, and within each category ordered the strains by size of the tagged ORF. Because of this reorganization, the six TAP-tagged proteins in each well of the multiplex extracts are of approximately equal abundance in extracts. Because the proteins are ordered by size within each abundance category, the six proteins in a given well also represent the maximal possible

Copyright © 2005 John Wiley & Sons, Ltd. 

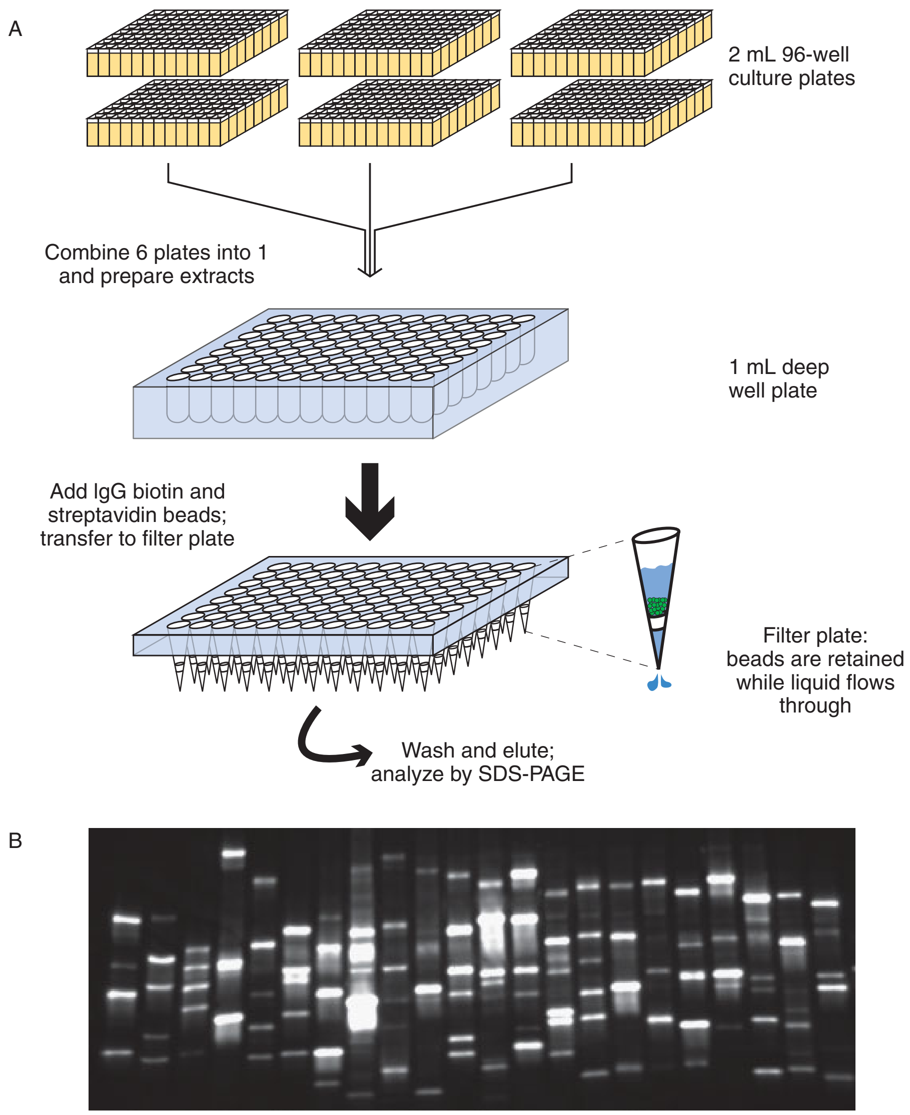

**Figure 3.** High-throughput 'multiplex' immunoprecipitations. (A) Schematic of process. Cultures from six 2 ml 96-well plates are pelleted, combined and used to prepare extracts. IgG biotin and streptavidin beads are added and allowed to bind, and then transferred to a filter plate that allows for retention of the beads. The beads are washed, and proteins are eluted and analysed by SDS-PAGE and immunoblot. (B) Example immunoblot of results of multiplex immunoprecipitation size distribution within the abundance category. This facilitates the resolution of the individual proteins in subsequent analysis by SDS-PAGE.

To test the feasibility of high-throughput immunoprecipitations, we prepared multiplex extracts and pulled down TAP-tagged proteins as described in the methods. In this particular experiment, the last column of the plate was left empty to provide space for controls necessary for a subsequent assay, so the theoretical maximum number of proteins pulled down in this reconstruction is 528 proteins. As shown in Figure 3B, we were successfully able to pull down a large number of proteins with this procedure.

To quantify the efficiency of the multiplex pulldown, we counted the number of distinct bands in each lane, and summed the total for this gel and the entire plate. We cannot discount the possibility that some of these bands may represent breakdown products or modified proteins; nevertheless, due to

Copyright © 2005 John Wiley & Sons, Ltd. 

the distinctness of the bands and the fact that they migrate at the appropriate molecular weight, it is likely that a significant proportion represent full-length proteins. It should be noted that because immunoprecipitation efficiency and potential post-translational modification or breakdown product formation is not identical for the six proteins in each lane, the multiplexing technique is not meant for comparisons between proteins within each lane, but rather for comparisons across different lanes (e.g. at different growth conditions or time points) for the same protein. The gel shown contains 22 lanes, or one-quarter of the plate, so the theoretical maximum number of immunoprecipitated proteins is 132. We are able to detect 111 distinct bands in this gel, or 84% of possible proteins. In immunoblots for the entire plate, we were able to detect 452 distinct bands, representing 86% of the 528 proteins possible. In separate experiments in which we immunoprecipitated TAP proteins from the collection individually, we were able to successfully pull down proteins with comparable efficiency (1070 proteins pulled down of 1334 attempts, or 79%). We conclude that immunoprecipitations in the multiplex format are an effective method of efficiently purifying TAP fusions from the collection.

### Discussion

In this paper, we have described the construction of two collections of yeast strains, both with C-terminal fusions of most ORFs in the yeast genome, one with the TAP tag and one with the GFP tag. The oligonucleotide primer set and the methods discussed could be used to efficiently construct a new collection with any desired C-terminal tag and in any desired genetic background. With the final refinements of our methods, a new collection could be constructed by a small team in a matter of months, depending on the degree of completeness and level of verification desired (expression analysis and reconstruction of our collections took several additional months). Furthermore, many of the methods described could be modified for use with other model organisms that support efficient homologous recombination.

We have confirmed that these collections do indeed represent a significant portion of the proteome, as we have confirmed expression of the ORF-tag fusions in individual transformants, either by immunoblot analysis for the TAP collection or by microscopic analysis for the GFP collection. Importantly, all fusions are under control of the native promoter, minimizing the potential for artifacts due to overexpression. We therefore believe that these collections will be useful tools in performing large-scale proteomic experiments. Indeed, in the course of confirming expression of the fusion proteins in these collections, we have been able to obtain valuable information about the absolute abundance of proteins (TAP collection) as well as their subcellular localization (GFP collection).

We wished to take proteomic analysis beyond being descriptive, so we developed methods to apply standard biochemical experiments in a parallel manner. We found that multiplexed immunoprecipitation could be performed efficiently from extracts derived from individual yeast cultures as small as 2 ml. This procedure should be easily modifiable in order to perform almost any extract-based assay in a parallel manner on the proteome.

These collections represent exciting possibilities for the future in a number of respects. First, it will be interesting to apply the descriptive methods outlined in this paper to experimental situations. One way in which cells can rapidly respond to environmental stimuli is to alter the localization, abundance or post-translational modification of proteins, sometimes without any change in transcriptional state. One could use the methods described in this paper to monitor these aspects of many proteins under various growth conditions or in response to environmental insults, either with the entire collections or by examining a specific subset or family of proteins.

Second, it will be exciting to see what types of biochemical and/or microscopic assays are applied in this high-throughput parallel manner. The ability to perform immunoprecipitations in a 'multiplex' format allows for efficient screening of the entire proteome with only a handful of experiments. Any number of post-translational modifications can be examined with minor modifications to this assay; it will be especially exciting to examine the dynamic nature of these modifications in response to environmental stimuli.

Furthermore, the ability to systematically select *MAT***a** haploid progeny from large-scale yeast crosses through the use of synthetic genetic array technology (SGA; Tong *et al.*, 2001) enables either

Copyright © 2005 John Wiley & Sons, Ltd. 

of these collections to be efficiently crossed to any desired genetic background. It should be noted that in previous studies, *HIS3* was used for *MAT***a**-specific selection in the 'magic marker' *MAT*$\alpha$ parent strain (Tong *et al*., 2001); because the TAP and GFP strains were constructed with the *HIS3* marker, an alternative marker (e.g. *LEU2*) must be used in any *MAT*$\alpha$ strains crossed to our collections for SGA assays. This permits the examination of how different mutations impact global localization, abundance or perhaps post-translational modifications, or could be used to sensitize the strain background to various chemical or environmental stimuli. One could also cross the TAP collection with a strain or strains containing an ORF tagged with a different epitope, thereby enabling high-throughput IP Western experiments to identify protein–protein interactions.

Worthy of mention is the ease and accessibility to high-throughput experimentation that these collections provide. Although we employed the use of robotics and other high-throughput equipment to construct these collections, it is possible to efficiently utilize these reagents with simply a handful of multi-channel pipettors and a 96-well pinning tool. We therefore hope that these collections will open the door to systematic proteomic analysis by a wider range of laboratories.

### Author's contributions

R.W.H., J.S.W. and E.K.O. conceived of the project. R.W.H. designed and oversaw synthesis of oligonucleotide primers. W.K.H. oversaw construction of the TAP and GFP collections. R.W.H., S.G., K.B., N.D., A.B., J.S.W. and E.K.O. assisted in construction of the TAP collection, and J.V.F. assisted in construction of the GFP collection. S.G. and K.B. performed expression analysis of the TAP collection. W.K.H. and J.V.F. performed expression analysis of the GFP collection. R.W.H. performed multiplex immunoprecipitation experiments with the TAP collection; D.D.W. provided data on individual immunoprecipitations. S.G., N.D., A.B. and D.D.W. performed reorganization of the TAP collection.

### Acknowledgements

We would like to thank Fran Sanchez for extraordinary organization of laboratory supplies and reagents used in these studies, and for aid in the synthesis of the oligonucleotides. David Ahern also assisted in oligo synthesis. Wendy Gilbert generously provided lyticase used in preparing extracts. Members of the O'Shea and Weissman labs provided helpful discussions, as well as critical review of this manuscript. R.W.H. was supported by a predoctoral fellowship from the National Science Foundation. S.G. and J.V.F. are recipients of the Ruth L. Kirschstein National Research Service Award. This work was supported by the Howard Hughes Medical Institute and the David and Lucile Packard Foundation (J.S.W. and E.K.O.).

### References

Aebersold R, Mann M. 2003. Mass spectrometry-based proteomics. *Nature* **422**: 198–207.

Brachat S, Dietrich FS, Voegeli S, *et al*. 2003. Reinvestigation of the *Saccharomyces cerevisiae* genome annotation by comparison to the genome of a related fungus, *Ashbya gossypii*. *Genome Biol* **4**: R45.

Brachmann BC, Davies A, Cost GJ, *et al*. 1998. Designer deletion strains derived from *Saccharomyces cerevisiae* S288C: a useful set of strains and plasmids for PCR-mediated gene disruption and other applications. *Yeast* **14**: 115–132.

Cliften P, Sudarsanam P, Desikan A, *et al*. 2003. Functional features in *Saccharomyces* genomes by phylogenetic footprinting. *Science* **301**: 71–76.

DeRisi JL, Iyer VR, Brown PO. 1997. Exploring the metabolic and genetic control of gene expression on a genomic scale. *Science* **278**: 680–686.

Diehn M, Eisen M, Botstein D, *et al*. 2000. Large-scale identification of secreted and membrane-associated gene products using DNA microarrays. *Nature Genet* **25**: 58–62.

Dolinski K, Balakrishnan R, Christie KR, *et al*. Saccharomyces Genome Database; **ftp://ftp.yeastgenome.org/yeast/** (accessed 17 April 2001).

Ghaemmaghami S, Huh W-K, Bower K, *et al*. 2003. Global analysis of protein expression in yeast. *Nature* **425**: 737–741.

Goffeau A, Coster F, Del Bino S, *et al*. 1997. The yeast genome directory. *Nature* **387**: (suppl): 1–105.

Haswell ES, O'Shea EK. 1999. An *in vitro* system recapitulates chromatin remodeling at the PHO5 promoter. *Mol Cell Biol* **19**: 2817–2827.

Huh W-K, Falvo JV, Gerke LC, *et al*. 2003. Global analysis of protein localization in budding yeast. *Nature* **425**: 686–691.

Kellis M, Patterson N, Endrizzi M, *et al*. 2003. Sequencing and comparison of yeast species to identify genes and regulatory elements. *Nature* **423**: 241–254.

Lockhart DJ, Dong H, Byrne MC, *et al*. 1996. Expression monitoring by hybridization to high-density oligonucleotide arrays. *Nature Biotechnol* **14**: 1675–1680.

Longtine MS, McKenzie A III, Demarini DJ, *et al*. 1998. Additional modules for versatile and economical PCR-based gene deletion and modification in *Saccharomyces cerevisiae*. *Yeast* **14**: 953–961.

MacBeath G, Schreiber SL. 2000. Printing proteins as microarrays for high-throughput function determination. *Science* **289**: 1760–1763.

Copyright © 2005 John Wiley & Sons, Ltd.

Martzen MR, McCraith SM, Spinelli SL, *et al*. 1999. A biochemical genomics approach for identifying genes by the activity of their products. *Science* **286**: 1153–1155.

Phizicky E, Bastiaens PI, Zhu H, *et al*. 2003. Protein analysis on a proteomic scale. *Nature* **422**: 208–215.

Rozen S, Skaletsky HJ. 1998. Primer3; **http://www-genome.wi.mit.edu/genome_software/other/primer3.html**

Schena M, Shalon D, Davis RW, *et al*. 1995. Quantitative monitoring of gene expression patterns with a complementary DNA microarray. *Science* **270**: 467–470.

Tong AH, Evangelista M, Parsons AB, *et al*. 2001. Systematic genetic analysis with ordered arrays of yeast deletion mutants. *Science* **294**: 2364–2368.

Zhu H, Bilgin M, Bangham R, *et al*. 2001. Global analysis of protein activities using proteome chips. *Science* **293**: 2101–2105.

Copyright © 2005 John Wiley & Sons, Ltd. 
